# ARHGAP1 Transported with Influenza Viral Genome Ensures Integrity of Viral Particle Surface through Efficient Budozone Formation

**DOI:** 10.1128/mbio.00721-22

**Published:** 2022-04-27

**Authors:** Takahiro Kuroki, Tomohisa Hatta, Tohru Natsume, Nobuaki Sakai, Akira Yagi, Kohsuke Kato, Kyosuke Nagata, Atsushi Kawaguchi

**Affiliations:** a Graduate School of Comprehensive Human Sciences, University of Tsukuba, Tsukuba, Japan; b Robotic Biology Institute Inc., Tokyo, Japan; c Molecular Profiling Research Center, National Institute for Advanced Industrial Science and Technology, Tokyo, Japan; d Department of Cell Physiology, Faculty of Medicine and Graduate School of Medicine, Hokkaido University, Sapporo, Japan; e R&D Group, Olympus Corporation, Hachioji, Japan; f Department of Infection Biology, Faculty of Medicine, University of Tsukuba, Tsukuba, Japan; g Transborder Medical Research Center, University of Tsukuba, Tsukuba, Japan; h Microbiology Research Center for Sustainability, University of Tsukuba, Tsukuba, Japan; McMaster University

**Keywords:** actin filament, influenza virus, recycling endosome, viral assembly

## Abstract

Influenza viral particles are assembled at the plasma membrane concomitantly with Rab11a-mediated endocytic transport of viral ribonucleoprotein complexes (vRNPs). The mechanism of spatiotemporal regulation of viral budozone formation and its regulatory molecules on the endocytic vesicles remain unclear. Here, we performed a proximity-based proteomics approach for Rab11a and found that ARHGAP1, a Rho GTPase-activating protein, is transported through the Rab11a-mediated apical transport of vRNP. ARHGAP1 stabilized actin filaments in infected cells for the lateral clustering of hemagglutinin (HA) molecules, a viral surface membrane protein, to the budozone. Disruption of the HA clustering results in the production of virions with low HA content, and such virions were less resistant to protease and had enhanced antigenicity, presumably because reduced clustering of viral membrane proteins exposes hidden surfaces. Collectively, these results demonstrate that Rab11a-mediated endocytic transport of ARHGAP1 with vRNPs stimulates budozone formation to ensure the integrity of virion surface required for viral survival.

## INTRODUCTION

Influenza A virus (IAV) has two major outer spike proteins, hemagglutinin (HA) and neuraminidase (NA), on its viral particles. HA is the major antigen for neutralizing antibodies and causes antigenic drift of the virus by human herd immunity, which is mostly directed against the globular head domain of HA densely packed on the viral particles (1–3). Little is yet known about the mechanism of the well-organized recruitment of viral spike proteins to the viral budding site to guarantee the structural integrity of the viral particles.

The IAV genome consists of eight single-stranded RNA segments of negative polarity. The viral genome exists as viral ribonucleoprotein complexes (vRNPs) by interacting with viral RNA-dependent RNA polymerases and nucleoprotein (NP). After nuclear transport of replicated vRNPs, the vRNPs are transported to the plasma membrane through Rab11a-positive recycling endosomes along microtubules for viral particle formation (4–7). The viral budding site, called the budozone, serves as a platform to concentrate the viral components at the plasma membrane. The assembly of the budozone is mediated by the formation of lipid rafts, which are membrane microdomains enriched in cholesterol/sphingolipids (8–10). HA and NA are intrinsically targeted to cholesterol-rich small lipid rafts and accumulate to the budozone through the clustering of small lipid rafts (11, 12). In contrast, viral protein M2 localizes to the boundary between the budozone and the bulk plasma membrane owing to its relatively short transmembrane domain, which prevents the insertion of M2 into ordered and tightly packed lipid rafts (13, 14). M2 has been proposed to mediate a viral membrane scission from the plasma membrane (15). Viral matrix protein M1 serves as an inner envelope-associated scaffold by interacting with both vRNPs and the cytoplasmic tails of HA and NA (16, 17).

Membrane trafficking mediated by Rab proteins is a major system to regulate the transport of cellular constituents between membrane organelles (18, 19). Rab proteins are small GTPases that are activated by a guanine nucleotide exchange reaction from GDP to GTP and bind to Rab effectors (20, 21). Through a recruitment of specific Rab effectors, Rab proteins function as molecular switches to deliver cargoes to their destinations by controlling the formation, motility, and fusion of endocytic vesicles (22). Endocytic recycling is carried out in either a direct manner, termed “fast recycling,” or “slow recycling,” that is mediated by endocytic recycling compartments (ERCs), which are a collection of tubular organelles around the microtubule-organizing center (MTOC) (23, 24). Rab11a-positive recycling endosomes (Rab11a REs) egress from ERCs and regulate the slow recycling pathway. Activated Rab11a interacts with its effector Rab11 family-interacting proteins (Rab11-FIPs). Rab11-FIPs are classified into two classes: class I (FIP1/RCP, FIP2, and FIP5/Rip11) and class II (FIP3 and FIP4). The Rab11-FIPs contain a highly conserved C-terminal Rab11-binding domain (RBD) and either a C2 domain (class I) or an EF-hand domain (class II) in the N terminus. FIPs regulate the dynamics of REs by bridging Rab11a and molecular motors (25). FIP2 and FIP5 interact with MyoVb and Kif3, respectively (26, 27), whereas FIP3 binds to dynein through Dyncli1 (28). Although the dynamics of endocytic transport mediated by REs are activated in infected cells (29), the function of FIPs in vRNP transport remains controversial. It has been reported that the overexpression of FIP RBDs dominant negatively disrupted the vRNP accumulation in ERCs, suggesting that the apical transport of vRNPs is mediated by FIPs (5). On the other hand, it has also been reported that vRNP binding to REs competes for the interaction of FIPs with Rab11a (29).

Using proximity-dependent biotin identification (BioID) screening, we identified cellular proteins involved in the endocytic transport and the organization of the actin cytoskeleton, including FIP2, FIP5, and ARHGAP1, a GTPase-activating protein (GAP) for the Rho GTPase family, as Rab11a-binding proteins in IAV-infected cells. ARHGAP1 was colocalized with vRNP in Rab11a REs, and their endocytic transport to the plasma membrane was dependent on FIP2 and FIP5. The vRNP transport promotes the clustering of HA to the viral budozone through the actin stabilization by ARHGAP1. We also found that knockdown (KD) of ARHGAP1 or FIP5 reduced viral titers and formed defective viral particles with low HA content that were less resistant to protease treatment and had enhanced antigenic potential. Collectively, we propose that the efficient budozone formation mediated by the Rab11a-ARHGAP1 pathway not only promotes the virus production but also guarantees the integrity of viral particle surface required for survival in the host environment.

## RESULTS

### FIP2- and FIP5-positive recycling endosomes regulate anterograde transport of vRNPs.

**FIG 1:**
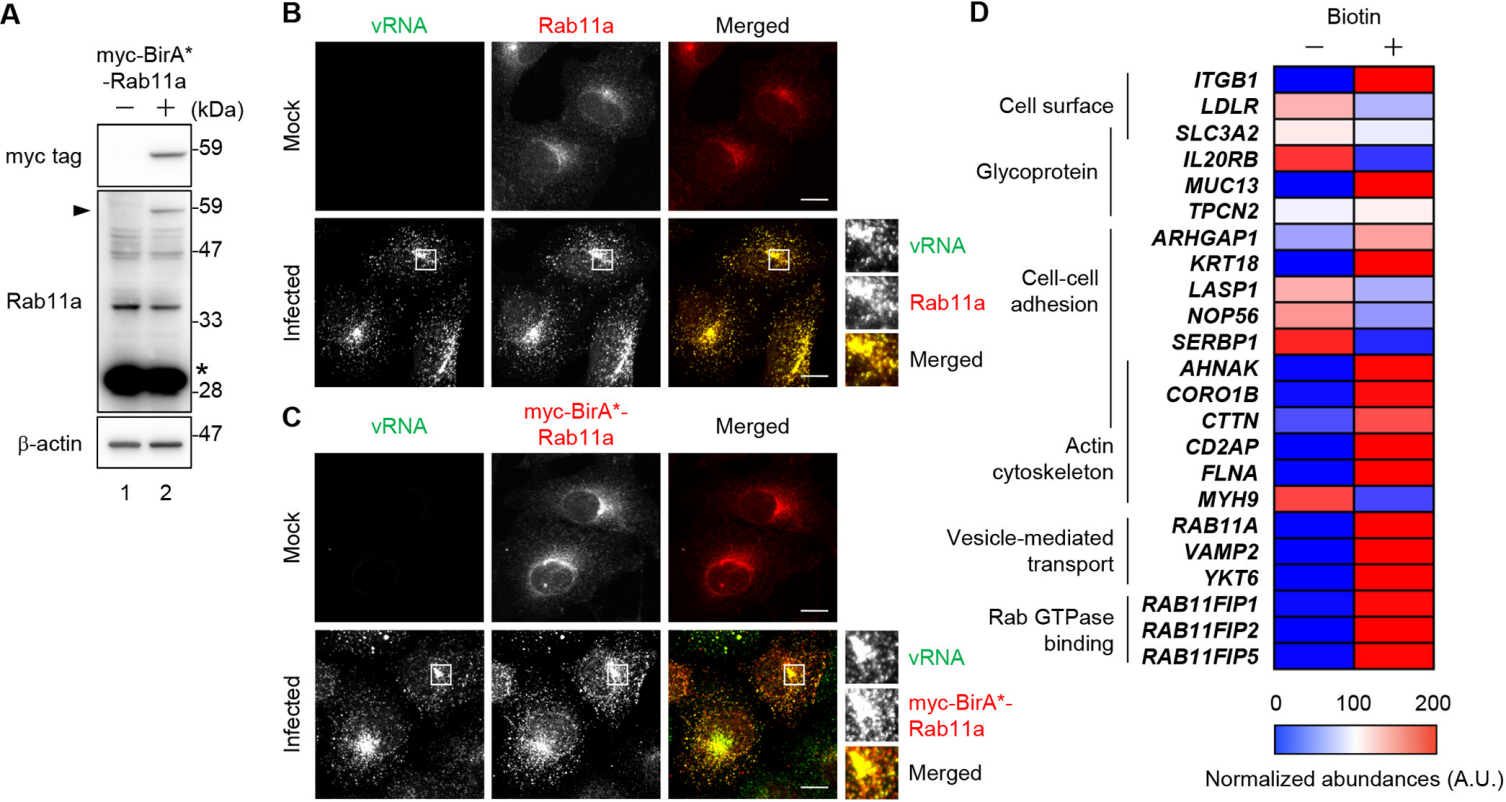
Identification of Rab11a-interacting proteins in IAV-infected cells. (A) Expression level of myc-BirA*-Rab11a. The cell lysates were obtained from A549 cells (lane 1) or myc-BirA*-Rab11a A549 cells (lane 2) and subjected to Western blot analyses with anti-myc tag, anti-Rab11a, and anti-β-actin antibodies. The asterisk and arrowhead indicate endogenous Rab11a and myc-BirA*-Rab11a, respectively. (B and C) At 4 h postinfection, A549 cells (B) and myc-BirA*-Rab11a A549 cells (C) were subjected to indirect immunofluorescence assays with anti-Rab11a (red in panel B) or anti-myc tag (red in panel C) antibodies, followed by FISH assays to detect seg7 vRNA (green). The enlarged panels show ERCs. Representative images from three independent experiments are shown. Scale bars, 10 μm. (D) Gene ontology analysis of Rab11a-interacting proteins in IAV-infected A549 cells was carried out using the DAVID database. The heatmap shows the normalized peptide abundance of identified proteins in LC-MS with or without the addition of biotin.

To identify the effector molecules that regulate the dynamics of Rab11a REs in infected cells, we carried out proximity-dependent biotin identification (BioID) assay with A549 cells stably expressing myc-tagged R118G-mutated BirA (BirA*)-fused Rab11a (Text S1). BirA is a bacterial biotin ligase that recognizes a specific amino acid sequence and links biotin to a lysine residue in the peptide by forming biotinoyl-5′-AMP (30). The promiscuous mutant BirA* has a lower affinity than wild-type BirA for biotinoyl-5′-AMP; thus, the active form of biotin transfers to lysine residues of proteins, which locate within a radius of 10 nm to a protein of interest fused to BirA* (31). The expression level of myc-BirA*-Rab11a (Fig. 1A, arrowhead) was less than that of endogenous Rab11a (Fig. 1A, asterisk). At 4 h postinfection, myc-BirA*-Rab11a was colocalized with the viral genome in the ERCs at the perinuclear regions as endogenous Rab11a was (Fig. 1B and C). The myc-BirA*-Rab11a A549 cells were infected with IAV at a multiplicity of infection (MOI) of 10 in the absence or presence of 50 μM biotin. At 8 h postinfection, the cell lysates were subjected to affinity purification with streptavidin beads. The purified biotinylated proteins were analyzed by liquid chromatography-mass spectrometry (LC-MS), and the identified proteins related to the cell surface, glycoproteins, cell-cell adhesion, actin cytoskeleton, vesicle-mediated transport, and Rab GTPase binding were shown (Fig. 1D and Table S1 in the supplemental material). We found that Rab11a interacts with class I Rab11-FIPs (FIP1, FIP2, and FIP5), but not class II Rab11-FIPs (FIP3 and FIP4), in infected cells (Fig. 1D). It was also observed that ARHGAP1, which functions as a GAP mainly for Cdc42 and RhoA, interacted with Rab11a in infected cells (Fig. 1D). It is reported that ARHGAP1 controls the assembly and disassembly of the actin filaments for transferrin uptake by regulating Rho GTPases (32–34).

10.1128/mbio.00721-22.7 TABLE S1 LC-MS analysis of Rab11a-interacting proteins in infected cells, related to Fig. 1D. For proteins identified in biotin-treated infected cells, gene symbol, gene description, experimental *q* values, sum posterior error probability (PEP) values, coverage in percentage, number of peptide spectrum matches (PSMs), number of unique peptides, number of amino acids (aa), molecular weight (MW) in kDa, calculated pI, and relative abundances in infected cells with or without biotin are shown. Download Table S1, XLSX file, 0.06 MB. Copyright © 2022 Kuroki et al. 2022 Kuroki et al. https://creativecommons.org/licenses/by/4.0/ This content is distributed under the terms of the Creative Commons Attribution 4.0 International license.

**FIG 2:**
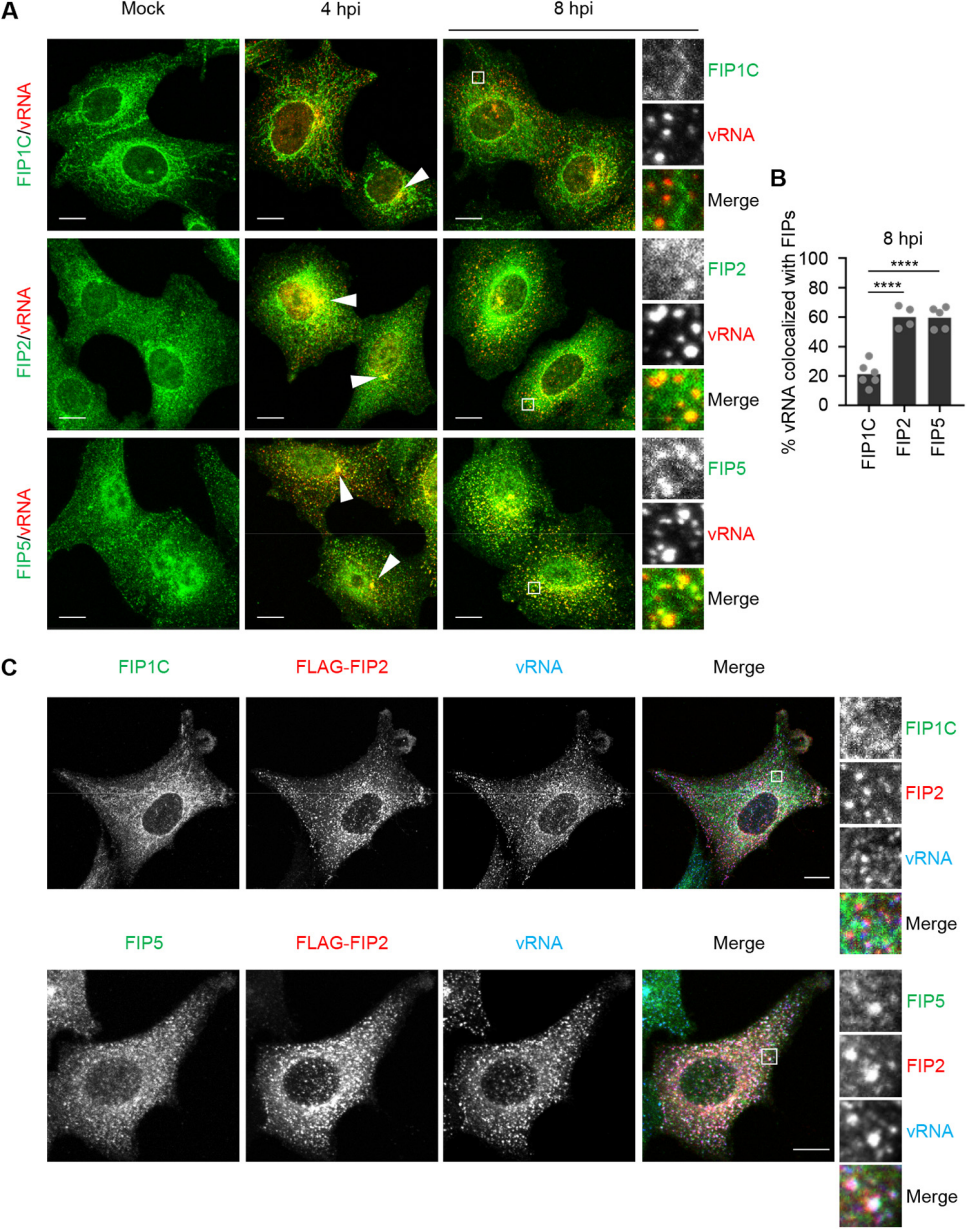
vRNPs colocalize with FIP2 and FIP5 but not with FIP1C. (A) A549 cells were infected with PR8 virus at an MOI of 10. At 4 and 8 h postinfection, the cells were subjected to indirect immunofluorescence assays with antibodies against class I FIPs (green), followed by FISH assays to detect seg7 vRNA (red). Arrowheads indicate ERCs. (B) Percentage of vRNA colocalized with FIPs in the cytoplasmic region of panel A at 8 h postinfection, excluding the region around ERC, was quantified using Imaris software (*n* = 4 to 6 cells/group). ****, *P* < 0.0001; one-way ANOVA with Tukey's test. (C) At 24 h posttransfection of FLAG-FIP2 (red), A549 cells were infected with PR8 virus at an MOI of 10. At 8 h postinfection, FIP1B (green, top), FIP5 (green, bottom), and seg7 vRNA (blue) were visualized by indirect immunofluorescence assays and FISH assays, respectively. Representative images from three independent experiments are shown. Scale bars, 10 μm (A and C).

To identify class I FIPs that are involved in the transport of vRNPs, we next examined the intracellular localization of viral RNA (vRNA) with FIP1C, FIP2, or FIP5 in infected A549 cells by indirect immunofluorescence assays and fluorescence *in situ* hybridization (FISH) assays (Fig. 2A). At 4 h postinfection, vRNA was colocalized with class I FIPs in the ERCs (Fig. 2A, arrowheads). However, upon the relocation of vRNA in punctate cytoplasmic structures for the apical transport at 8 h postinfection, the dispersed vRNA was colocalized with FIP2 and FIP5 only, but not with FIP1C (Fig. 2A and B). Furthermore, transiently expressed FLAG-FIP2 was colocalized with FIP5, but not with FIP1C (Fig. 2C).

**FIG 3:**
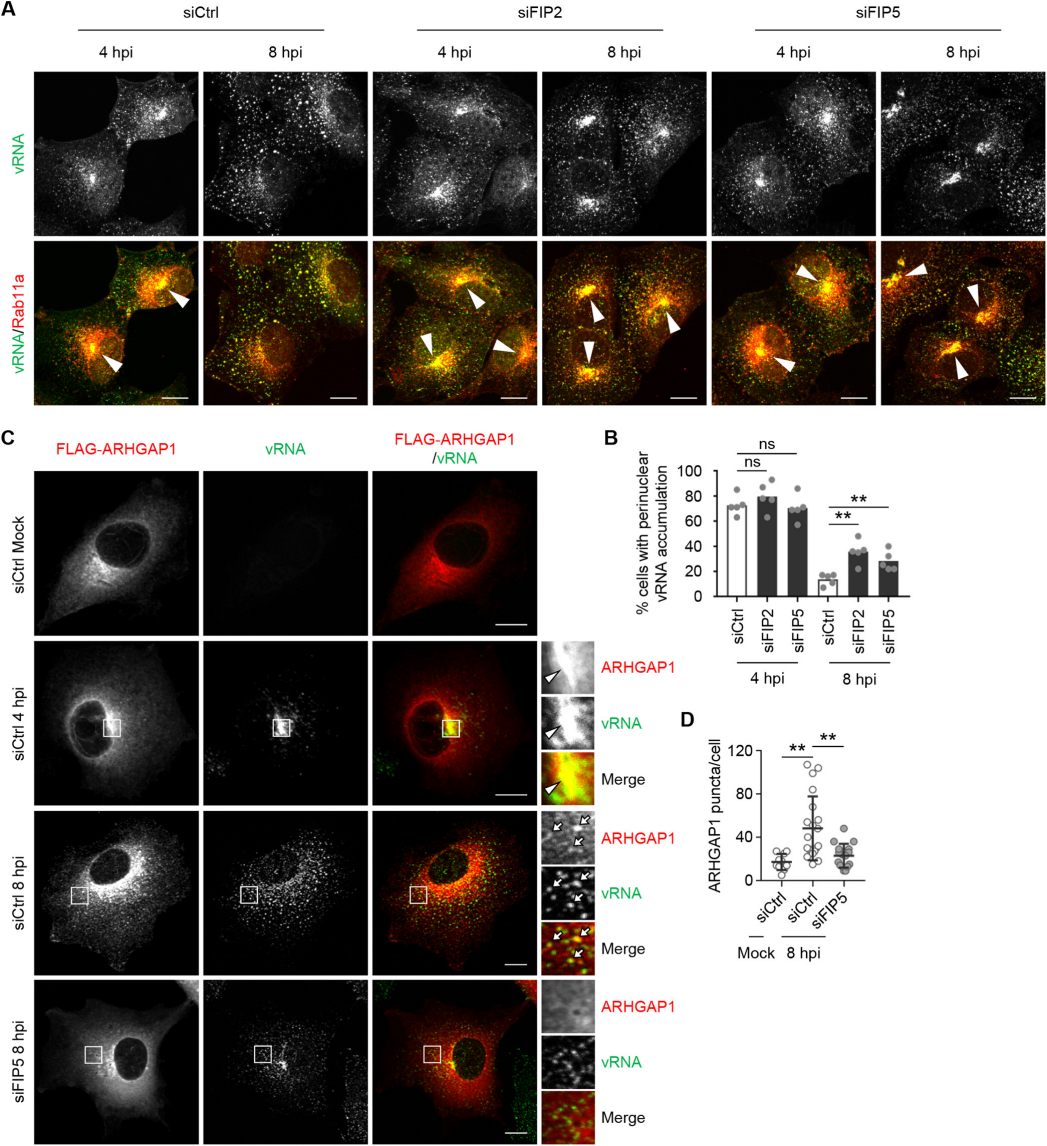
vRNPs reach to the plasma membrane together with ARHGAP1 in an FIP5-dependent manner. (A) A549 cells were transfected with scrambled (siCtrl), siFIP2 1, or siFIP5 1. At 48 h posttransfection, the cells were infected with PR8 virus at an MOI of 10. At 4 and 8 h postinfection, Rab11a (red) and seg7 vRNA (green) were visualized by indirect immunofluorescence assays and FISH assays. The arrowheads indicate ERCs. Representative images from three independent experiments are shown. (B) Percentage of cells showing the perinuclear accumulation of vRNA signal in panel A was measured (*n* = 5 fields with 60 to 113 cells/group). **, *P* < 0.01; one-way ANOVA with Tukey's test; ns, not significant. (C) FLAG-ARHGAP1-expressing A549 cells were transfected with scrambled (siCtrl) or siFIP5 1. At 48 h posttransfection, the cells were infected with PR8 virus at an MOI of 10. At 4 and 8 h postinfection, seg7 vRNA (green) and FLAG-ARHGAP1 (red) were visualized by FISH assays and indirect immunofluorescence assays with anti-FLAG antibody. Representative images from three independent experiments are shown. The arrowheads and arrows indicate ERCs and cytoplasmic punctate signals positive for FLAG-ARHGAP1 and vRNA, respectively. (D) The number of cytoplasmic FLAG-ARHGAP1 puncta in control or FIP5 KD cells of panel C were calculated using Imaris software (siCtrl mock, *n* = 10 cells; siCtrl at l 8 hpi, *n* = 20 cells; siFIP5 at 8 hpi, *n* = 16 cells). Results are means ± SDs. **, *P* < 0.01; one-way ANOVA with Tukey's test. Scale bars, 10 μm (A and C).

At 48 h posttransfection of scrambled small interfering RNA (siRNA) (siCtrl) or siRNA for *RAB11FIP2* (siFIP2) or *RAB11FIP5* (siFIP5), the expression levels of FIP2 and FIP5 in the KD cells had decreased to less than 10% of that in the control cells, respectively (Fig. S1A and B). Note that the expression level of NP was not changed by FIP KD (Fig. S1C). We found that the extent of vRNP accumulation at the ERCs in FIP2 KD or FIP5 KD cells was comparable to that in the control cells at 4 h postinfection (Fig. 3A and B). However, vRNPs remained in the ERCs even at 8 h postinfection in FIP2 KD or FIP5 KD cells (Fig. 3A and B and Fig. S2A and B), suggesting that FIP2 and FIP5 cooperatively regulate the apical transport of vRNPs by Rab11a REs emanated from ERCs. We also found that FLAG-ARHGAP1 localized with vRNA in the ERCs at 4 h postinfection (Fig. 3C, arrowheads). Furthermore, at 8 h postinfection, FLAG-ARHGAP1 signals translocated to the punctate cytoplasmic structures with vRNA in an FIP5-dependent manner (Fig. 3C, arrows, and Fig. 3D), indicating that ARHGAP1 is transported to the plasma membrane with vRNA through Rab11a REs.

10.1128/mbio.00721-22.1 FIG S1 Knockdown of FIP2 and FIP5. (A and B) A549 cells were transfected with scrambled siRNA (siCtrl), siFIP2 1, and siFIP2 2 (A) or siFIP5 1 and siFIP5 2 (B). At 48 h posttransfection, the expression levels of FIP2 (A) or FIP5 (B) were examined. (C) A549 cells were transfected with scrambled siRNA (siCtrl), siFIP2 1, or siFIP5 1. At 48 h posttransfection, the cells were infected with PR8 virus at an MOI of 10. At 3 and 5 h postinfection, the expressions of viral NP protein and β-actin were examined. Asterisks indicate nonspecific signals (A and C). Download FIG S1, TIF file, 0.1 MB. Copyright © 2022 Kuroki et al. 2022 Kuroki et al. https://creativecommons.org/licenses/by/4.0/ This content is distributed under the terms of the Creative Commons Attribution 4.0 International license.

10.1128/mbio.00721-22.2 FIG S2 Intracellular localization of vRNA in FIP2 or FIP5 KD cells. (A) A549 cells transfected with scrambled siRNA (siCtrl), siFIP2 2, or siFIP5 2 were infected with PR8 virus at a MOI of 10. At 8 h postinfection, Rab11a (red) and seg7 vRNA (green) were visualized as described in Fig. 3A. The arrowheads indicate ERCs. Representative images from three independent experiments are shown. Scale bar, 10 μm. (B) The percentage of cells showing the perinuclear accumulation of vRNA signal in panel A was measured (*n* = 5 fields with 57 to 105 cells/group). **, *P* < 0.01; ***, *P* < 0.001; one-way ANOVA with Tukey's test. Download FIG S2, TIF file, 0.8 MB. Copyright © 2022 Kuroki et al. 2022 Kuroki et al. https://creativecommons.org/licenses/by/4.0/ This content is distributed under the terms of the Creative Commons Attribution 4.0 International license.

### Formation of the budozone requires actin filaments.

**FIG 4:**
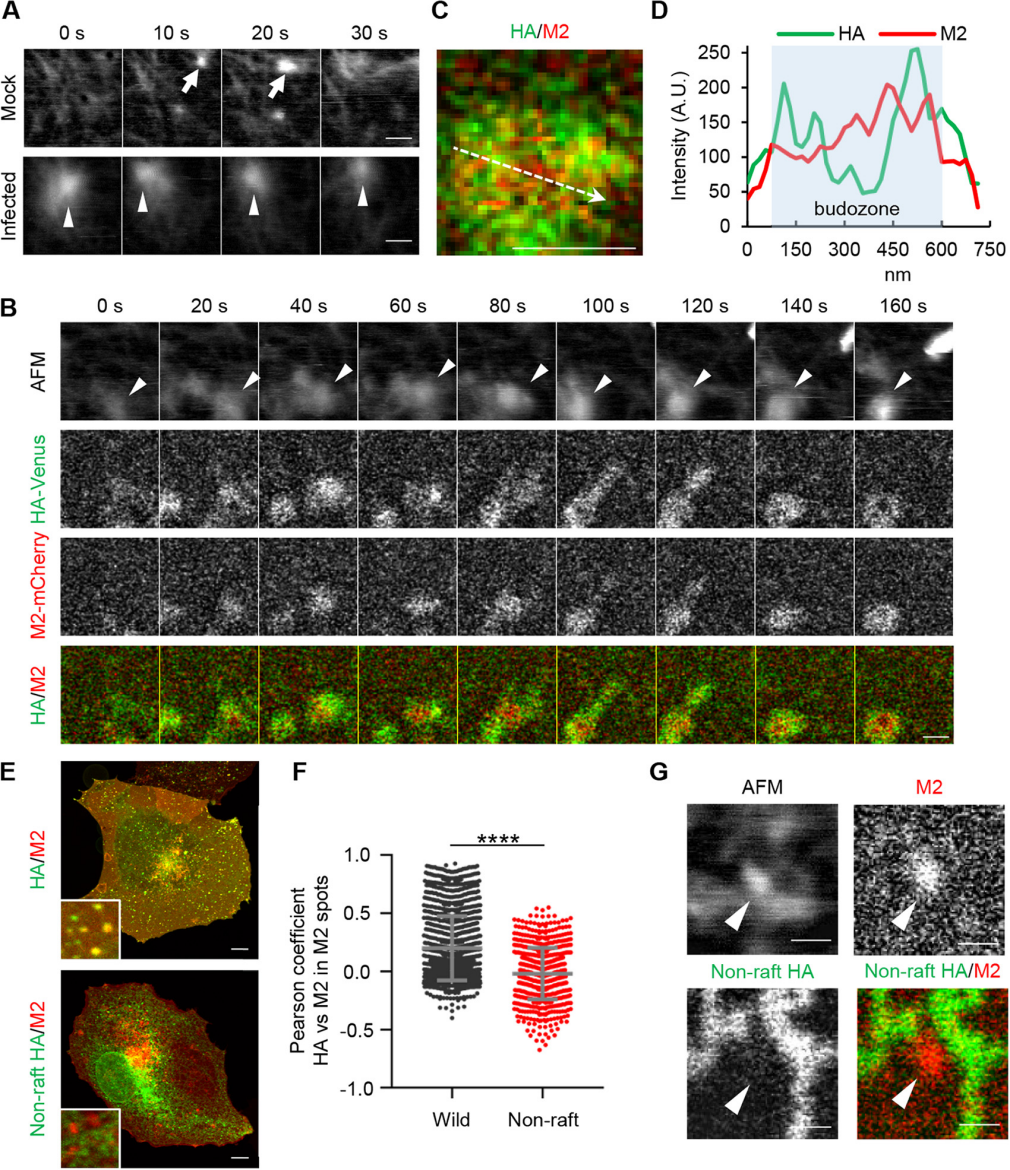
HS-AFM/fluorescence imaging of budozone-like protrusions. (A) COS-7 cells were infected with PR8 virus at an MOI of 10. At 12 h postinfection, the cells were subjected to time-lapse AFM imaging. (B) At 1 h posttransfection of plasmids expressing HA-Venus (green) and M2-mCherry (red), COS-7 cells were infected with PR8 virus. At 12 h postinfection, HS-AFM/fluorescence imaging studies were performed. (C) Enlarged image at 150 s after the observation in panel B. (D) Relative pixel intensities of M2-mCherry (red) and HA-Venus (green), along with the arrow, are shown. (E) Infected COS-7 cells expressing M2-mCherry (red) and nonraft HA-Venus (green) were observed by confocal microscopy. (F) The Pearson coefficient between the HA signal and M2 signal in each M2 spot is expressed. (G) Infected COS-7 cells expressing M2-mCherry (red) and either wild-type or nonraft HA-Venus (green) were observed by HS-AFM/fluorescence microscopy. Results are means ± SDs. ****, *P* < 0.0001; two-tailed Student's *t* test. The arrows and arrowheads in panels A, B, and G indicate membrane ruffles and budozone-like protrusions, respectively. Scale bars, 500 nm (A, B, C, and G) or 10 μm (E).

The assembly and membrane protrusion of the viral budozone are coupled with the clustering of lipid rafts in which viral membrane proteins are embedded (35, 36). Thus, we examined the dynamics of budozone formation by a high-speed atomic force/fluorescence microscopy imaging system (BIXAM). At 1 h posttransfection of HA-Venus and M2-mCherry, the cells were infected with IAV at an MOI of 10 for 12 h. The budozone-like protrusions were approximately 500 nm in diameter as previously observed by transmission electron microscopy (TEM) imaging (Fig. 4A, arrowheads) (3, 37). Further, the protrusions colocalized with HA-Venus and M2-mCherry were persistently observed over a few minutes (Fig. 4B, arrowheads). It was clearly distinct from membrane ruffles, which were less than 400 nm in diameter and disappeared within tens of seconds (Fig. 4A, arrows). In the budozone-like protrusions, M2-mCherry was surrounded by HA-Venus in a ringlike pattern (Fig. 4B to D). This is in good agreement with the fact that M2 mediates the pinching off of virus particles from the plasma membrane (15). In contrast, a mutant HA harboring alanine substitutions (I532A, Y533A, and S534A) in the cholesterol-binding domain, referred to as nonraft HA (38, 39), hardly colocalized with M2 in the protruding structures (Fig. 4E to G). Although we have not excluded the possibility that nonraft HA is defective in the interaction with other structural proteins such as M1 and M2, it is possible that the accumulation of HA in the viral budozone is mediated by lipid rafts at the plasma membrane through the binding of HA to cholesterol.

**FIG 5:**
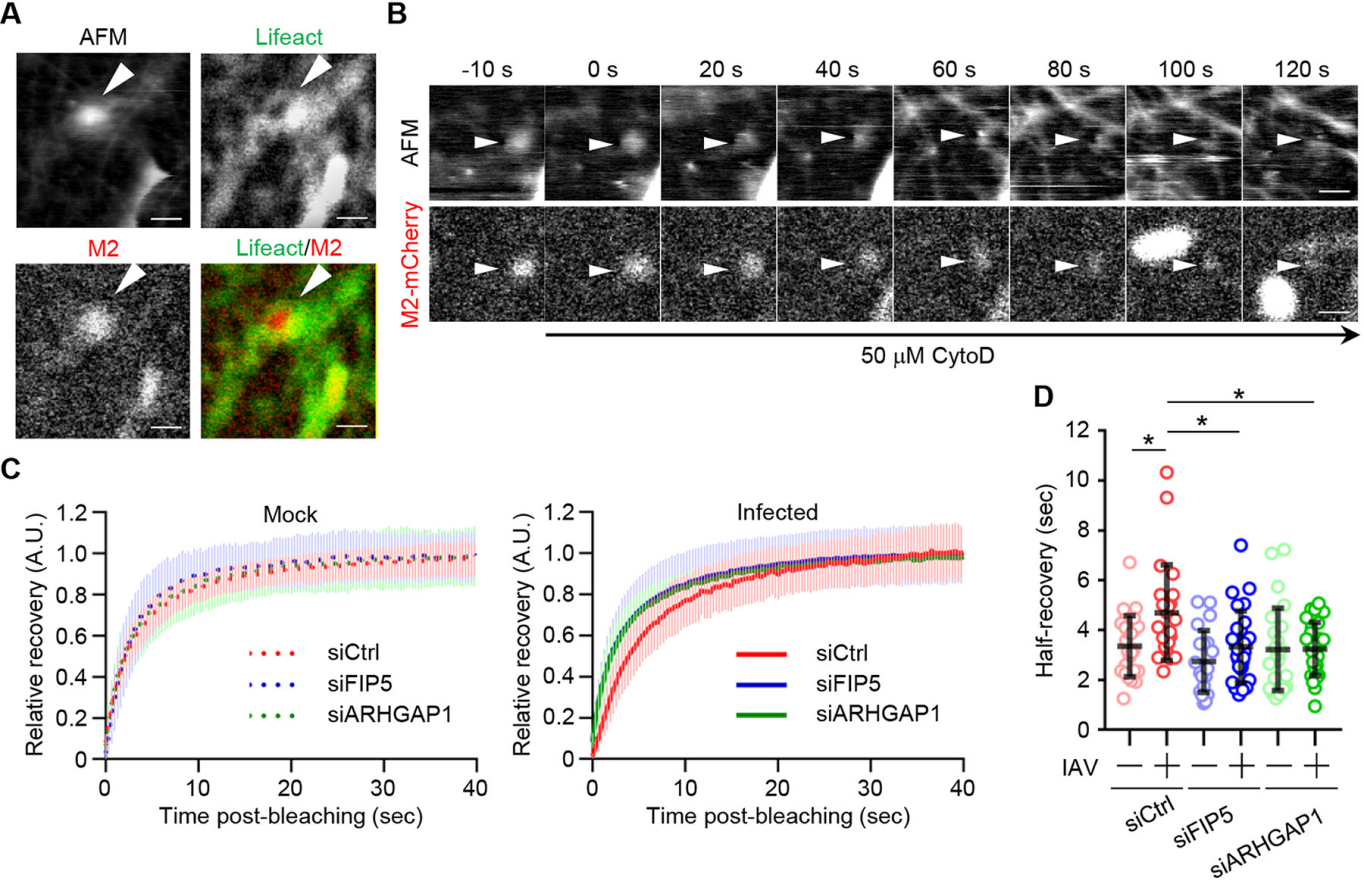
Viral budozone formation requires actin filaments stabilized by FIP5 and ARHGAP1. (A and B) COS-7 cells expressing Lifeact-TagGFP2 (green) and M2-mCherry (red) (A) or M2-mCherry alone (B) were infected with PR8 virus. At 12 h postinfection, HS-AFM/fluorescence imaging studies were performed in the absence (A) or presence (B) of 50 μM cytochalasin D (CytoD). The arrowheads indicate budozone-like protrusions. Scale bars, 500 nm. (C and D) At 12 h postinfection, the mobility of Lifeact-TagGFP2 in A549 cells transfected with scrambled (siCtrl), siFIP5 1, or siARHGAP1 1 was analyzed by FRAP assays. (C) Relative fluorescence intensities of the bleached regions against the nonbleached regions are shown. (D) The mean half-recovery time was calculated using GraphPad Prism software (*n* = 21 to 28 cells/group). Results are means ± SDs. *, *P* < 0.05; one-way ANOVA with Tukey's test.

It is proposed that the formation of lipid rafts requires the lateral mobility of membrane molecules at the plasma membrane through cortical actin filaments (40, 41). We found that actin filaments visualized by expressing Lifeact-TagGFP2 associate with the budozone-like protrusions (Fig. 5A, arrowheads). Further, the budozone was rapidly made to disappear by addition of 50 μM cytochalasin D (CytoD), a potent actin polymerization inhibitor (Fig. 5B, arrowheads). These results indicated that actin filaments function as a scaffold to assemble the viral budozone at the plasma membrane. We also tested the dynamics of actin filaments in infected cells expressing Lifeact-TagGFP2 by fluorescence recovery after photobleaching (FRAP) assays. The mean half-recovery time of Lifeact-TagGFP2 was 5.60 s for infected cells, but 3.97 s for uninfected cells (Fig. 5C and D and Fig. S2A), indicating that actin filaments are relatively stabilized by IAV infection. We also examined the actin dynamics by FRAP assays in FIP5 KD (siFIP5) or ARHGAP1 KD cells (siARHGAP1). The expression levels of ARHGAP1 in the KD cells decreased to about 20% of those in the control cells (Fig. S3A). The delay of the mean half-recovery time of Lifeact-TagGFP2 upon IAV infection was not observed in FIP5 KD or ARHGAP1 KD cells (Fig. 5C and D and Fig. S3B to D). However, this was not the case for FIP1 KD cells (Fig. S4A to C). It is likely that actin filaments are stabilized by ARHGAP1 transported to the plasma membrane through FIP5-regulated REs in infected cells.

10.1128/mbio.00721-22.3 FIG S3 FRAP assays in control, FIP5 KD, and ARHGAP1 KD cells. (A) A549 cells were transfected with scrambled siRNA (siCtrl), siARHGAP1 1, and siARHGAP1 2. At 48 h posttransfection, the expression level of ARHGAP1 was examined. (B) At 12 h postinfection, the mobility of Lifeact-TagGFP2 in A549 cells transfected with scrambled siRNA (siCtrl), siFIP5 1, and siARHGAP1 1 was analyzed by FRAP assays, related to Fig. 5C and D. Photobleached rectangular regions are indicated. Scale bars, 2 μm. (C and D) At 12 postinfection, the mobility of Lifeact-TagGFP2 in A549 cells transfected with scrambled siRNA (siCtrl), siFIP5 2, and siARHGAP1 2 was analyzed by FRAP assays, and the relative recovery curve and the mean half-recovery time were obtained as described in Fig. 5C and D (*n* = 21 to 43 cells/group). Results are means ± SDs. ****, *P* < 0.0001; one-way ANOVA with Tukey's test. Download FIG S3, TIF file, 1.1 MB. Copyright © 2022 Kuroki et al. 2022 Kuroki et al. https://creativecommons.org/licenses/by/4.0/ This content is distributed under the terms of the Creative Commons Attribution 4.0 International license.

10.1128/mbio.00721-22.4 FIG S4 FRAP assays in control and FIP1 KD cells. (A) A549 cells were transfected with scrambled (siCtrl) or FIP1 siRNA. At 48 h posttransfection, cell lysates were subjected to Western blot assays with anti-FIP1 and anti-β-actin antibodies. (B and C) At 12 h postinfection, the mobility of Lifeact-TagGFP2 in control and FIP1 KD cells was analyzed by FRAP assays, and the relative recovery curve and the mean half-recovery time were obtained as described in Fig. 5C and D (*n* = 20 to 24 cells/group). Results are means ± SDs. Download FIG S4, TIF file, 0.3 MB. Copyright © 2022 Kuroki et al. 2022 Kuroki et al. https://creativecommons.org/licenses/by/4.0/ This content is distributed under the terms of the Creative Commons Attribution 4.0 International license.

### Efficient budozone formation confers protease resistance and reduced antigenicity to the viral particles.

**FIG 6:**
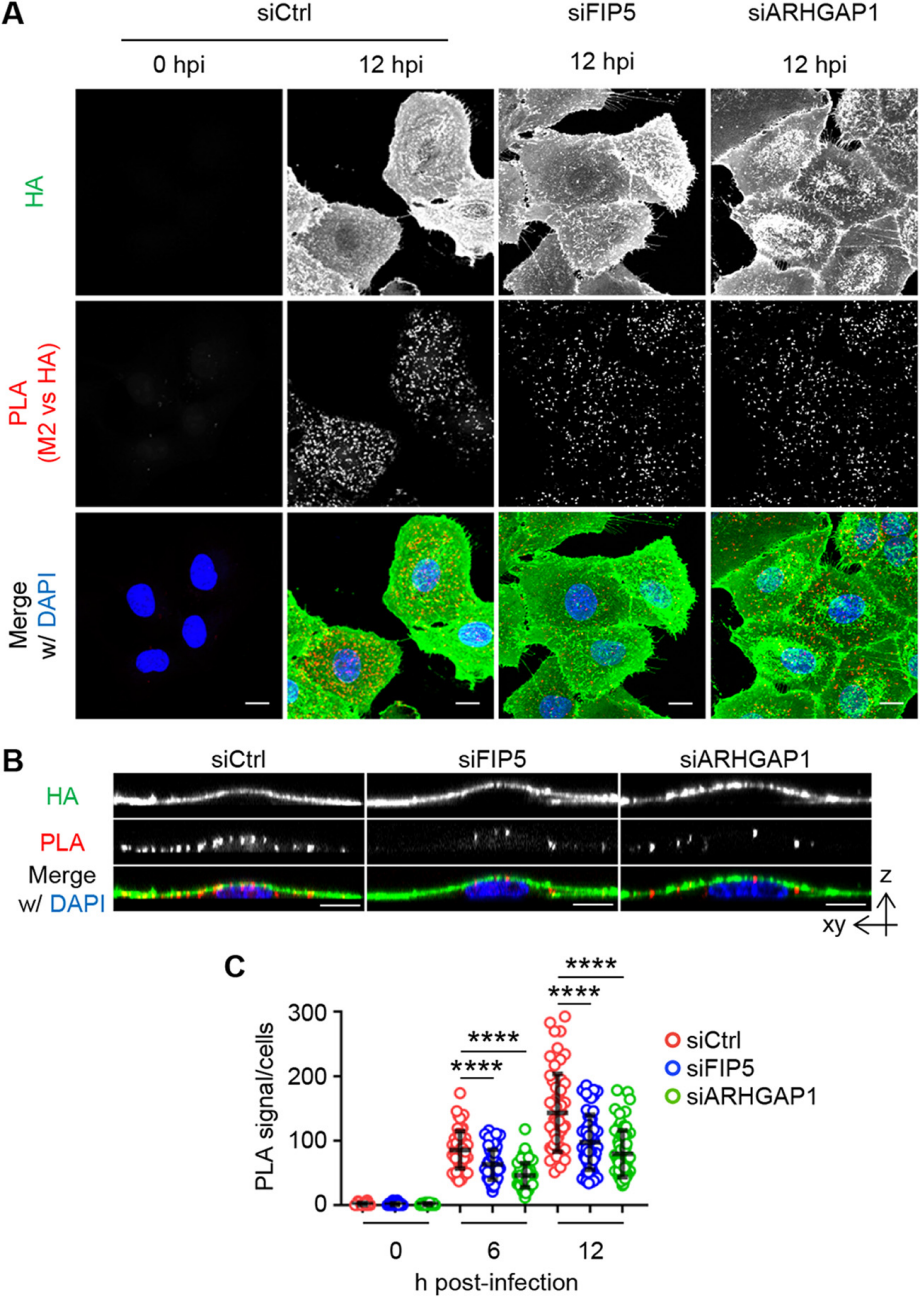
FIP5 and ARHGAP1 promote viral budozone formation. (A) A549 cells transfected with scrambled (siCtrl), siFIP5 1, or siARHGAP1 1 were infected with PR8 virus at an MOI of 10. At 6 and 12 h postinfection, the cells were subjected to PLA assays with anti-HA and anti-M2 antibodies (PLA signals, red). HA (green) and DNA (blue) were counterstained with anti-mouse IgG conjugated with Alexa 488 and DAPI (4′,6-diamidino-2-phenylindole). (B) The vertical section images from the z-stack series of panel A were reconstructed. Representative images from three independent experiments are shown. (C) The number of PLA signals per cell in panel A were analyzed by Imaris software (means ± SDs; *n* = 50 to 63 cells/group). ****, *P* < 0.0001; one-way ANOVA with Tukey's test.

**FIG 7:**
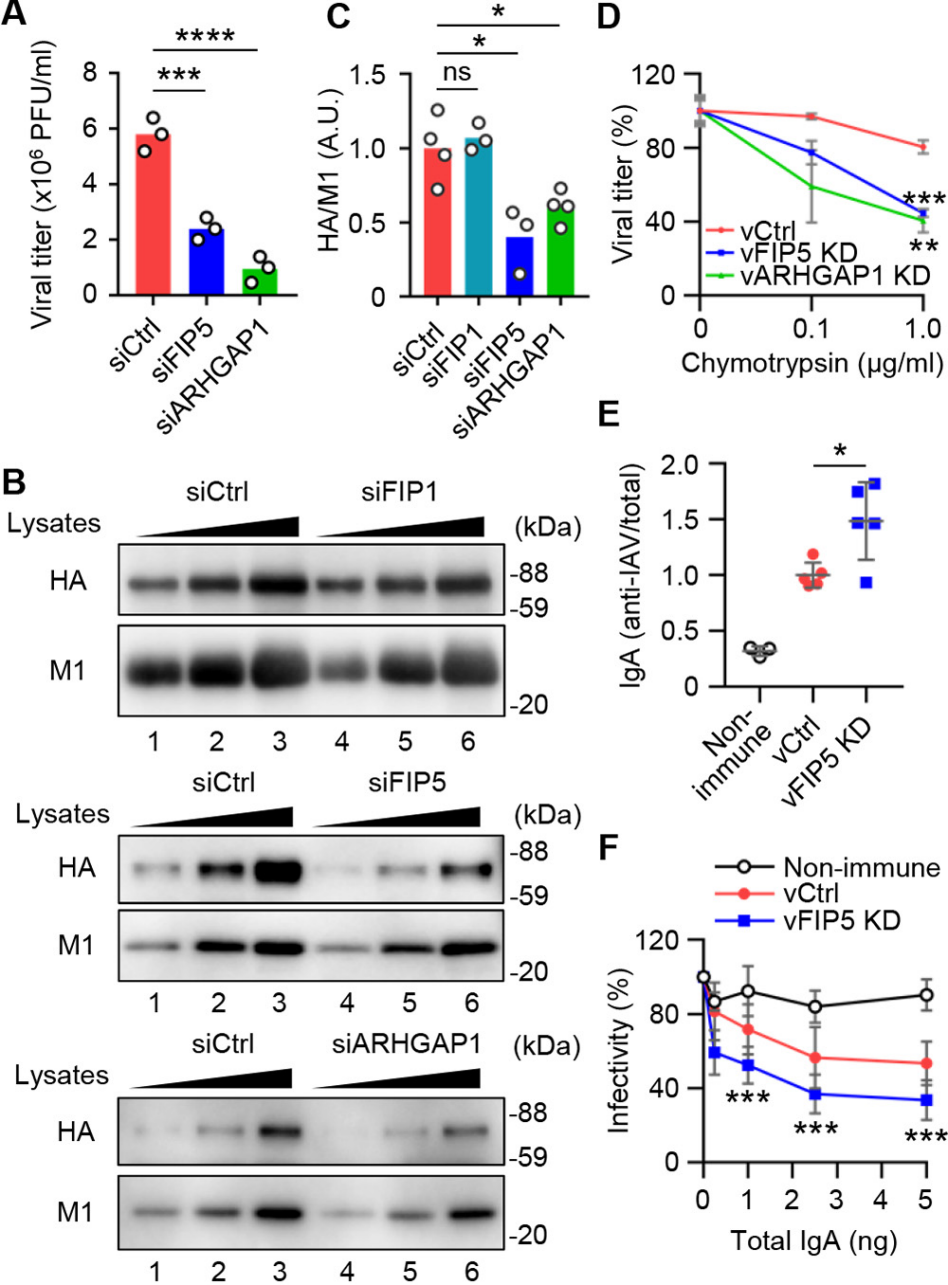
FIP5 and ARHGAP1 promote the production of stable progeny viral particles with reduced antigenicity. (A) A549 cells transfected with scrambled (siCtrl), siFIP5 1, or siARHGAP1 1 were infected with WSN virus at an MOI of 0.1. At 48 h postinfection, the viral titers in the culture supernatants were examined by plaque assays (*n* = 3, independent experiments). (B and C) Control, FIP1KD, FIP5 KD, and ARHGAP1 KD A549 cells were infected with WSN virus at an MOI of 0.1. At 48 h postinfection, the culture supernatants were ultracentrifuged through a 20% (wt/vol) sucrose cushion at 78,400 × *g* for 1.5 h at 4°C. (B) The different volumes (2.5, 5, and 10 μL) of purified viral particles were analyzed by SDS-PAGE followed by Western blot analyses with anti-HA and anti-M1 antibodies. (C) The relative amount of HA against M1 is shown (*n* = 3 or 4; independent experiments). (D) Virions produced from the control (vCtrl), FIP5 KD (vFIP5 KD), and ARHGAP1 KD (vARHGAP1) A549 cells were treated with chymotrypsin at a final concentration of 0, 0.1, or 1 μg/mL at 37°C for 3 h and then subjected to plaque assays. (E) The levels of IgA antibodies in nasal washes obtained from mice immunized with vCtrl or vFIP5 KD were determined by ELISA. The ratios of IAV-specific IgA against total IgA are shown (*n* = 3 to 5 mice per group). (F) PR8 virus was incubated with different concentrations of total IgA in nasal washes obtained from mice immunized with vCtrl or vFIP5 KD for 2 h. Each viral titer was determined by focus-forming assays with MDCK cells. The percentage of infected cells compared with the sample without nasal washes was represented as percentage of infectivity. *, *P* < 0.05; **, *P* < 0.01; ***, *P* < 0.001; ****, *P* < 0.0001; one-way ANOVA with Tukey's test (A, C, D, and F) or two-tailed Student's *t* test (E). Results are means ± SDs (D to F).

We next examined the formation of the viral budozone in FIP5 KD and ARHGAP1 KD cells by *in situ* proximity ligation assays (PLAs) to detect the close proximity between HA and M2. The number of PLA signals between HA and M2 decreased to 68% of that of the control cells by FIP5 KD and to 56% by ARHGAP1 KD without impairing the apical transport of HA (Fig. 6A to C and Fig. S5A and B). These results indicated that the FIP5-mediated transport of ARHGAP1, together with vRNP, promotes the budozone formation at the plasma membrane. Note that newly synthesized HA and NA proteins are transported to the apical plasma membrane through the *trans*-Golgi network (TGN) in a Rab11a-independent manner (39, 42–44). It is also reported that newly synthesized M2 protein is apically transported mainly by the Golgi apparatus (45) and in part by Rab11a-dependent endocytic transport (15). However, we found that FIP5 KD and ARHGAP1 KD had little effect on the surface M2 protein level (Fig. S5C and D). In line with the inhibition of budozone formation, the viral titers were reduced to 41% of the control cells by FIP5 KD and to 16% by ARHGAP1 KD (Fig. 7A). To quantitatively analyze the amount of HA on virions, virions were purified from the culture supernatants of infected cells treated with siFIP5 or siARHGAP1 by the ultracentrifugation through a sucrose cushion (Fig. 7B and C). The protein ratios of HA relative to M1, which is a scaffold protein of viral particles (16, 46–48), in purified virions obtained from FIP5 KD or ARHGAP1 KD cells were reduced to around 40% to 60% of those from control cells, respectively (Fig. 7B and C). Note that the HA/M1 ratio of virions obtained from FIP1 KD cells was unchanged (Fig. 7B and C). Note that TEM observations indicated that the purified virions from FIP5 KD cells and ARHGAP1 KD cells did not differ in the diameter of viral particles but contained fewer viral spike proteins than those from the control cells (Fig. S6A to C).

10.1128/mbio.00721-22.5 FIG S5 PLA assays between HA and M2 in FIP5 KD or ARHGAP1 KD cells. (A) A549 cells transfected with scrambled siRNA (siCtrl), siFIP5 2, or siARHGAP1 2 were infected with PR8 virus at a MOI of 10. At 12 h postinfection, the cells were subjected to PLA assays with anti-HA and anti-M2 antibodies (PLA signals, red). HA (green) and DNA (blue) were counterstained with anti-mouse IgG conjugated with Alexa 488 and DAPI. Bottom panels show the reconstructed vertical section images. Representative images from three independent experiments are shown. (B) The number of PLA signals per cell in panel A were analyzed by Imaris software (means ± SDs; *n* = 24 to 30 cells/group). ****, *P* < 0.0001; one-way ANOVA with Tukey's test. (C) Control, FIP5 KD, and ARHGAP1 KD A549cells were infected with PR8 virus at an MOI of 10. At 12 h postinfection, M2 (green) localized on the cell surface was visualized by indirect immunofluorescence assays with anti-M2 antibody. DNA (blue) was stained with DAPI. Bottom panels show the reconstructed vertical section images. Representative images from three independent experiments are shown. (D) The intensity of M2 signals of panel C was analyzed by Imaris software (means ± SDs; *n* = 16 to 21 cells/group). Scale bars, 10 μm (A and C). Download FIG S5, TIF file, 1.0 MB. Copyright © 2022 Kuroki et al. 2022 Kuroki et al. https://creativecommons.org/licenses/by/4.0/ This content is distributed under the terms of the Creative Commons Attribution 4.0 International license.

10.1128/mbio.00721-22.6 FIG S6 TEM analysis of virions purified from FIP5 KD and ARHGAP1 KD cells. (A) Control, FIP5 KD, and ARHGAP1 KD A549 cells were infected with IAV at MOI of 0.1. At 48 h postinfection, the culture supernatants were ultracentrifuged on a 20% (wt/vol) sucrose cushion at 78,400 × *g* for 1.5 h at 4°C. The purified viral particles were absorbed on the copper mesh grids, negatively stained, and then observed by TEM. Representative images from three independent experiments are shown. Scale bars, 50 nm. (B and C) The diameter of viral particles (B) and the number of viral spike proteins on viral particles (C) were measured (means ± SDs; *n* = 13 to 19 viral particles/group). *, *P* < 0.05; ****, *P* < 0.0001; one-way ANOVA with Tukey's test. Download FIG S6, TIF file, 0.4 MB. Copyright © 2022 Kuroki et al. 2022 Kuroki et al. https://creativecommons.org/licenses/by/4.0/ This content is distributed under the terms of the Creative Commons Attribution 4.0 International license.

The intact viral particles are relatively resistant to digestion by proteases, and their epitopes are concentrated on the exposed surfaces, including the globular head domain, possibly due to steric hindrance of clustered HA. Thus, we assumed that the efficient HA packing on viral particles is important for the stability and the antigenic potential of viral particles. To address this possibility, the progeny viruses produced from control cells (vCtrl), FIP5 KD cells (vFIP5 KD), or ARHGAP1 KD cells (vARHGAP1 KD) were treated with chymotrypsin at a final concentration of 0, 0.1, or 1 μg/mL. After incubation at 37°C for 3 h, the viral titer was determined by plaque assays. Although the progeny viruses from the control cells were resistant to the protease treatment, the infectivity of progeny viruses from FIP5 KD or ARHGAP1 KD cells was reduced to around 40% of that of the control cells by addition of 1 μg/mL chymotrypsin (Fig. 7D). We next examined the antigenic potential of progeny viral particles produced from control cells and FIP5 KD cells (Fig. 7E). Mice were intranasally immunized with formalin-inactivated virions containing 1 μg of HA produced from control or FIP5 KD cells, and then the ratio of IAV-specific IgA against total IgA in nasal washes was quantified by enzyme-linked immunosorbent assay (ELISA). Because the viral titer was largely reduced in ARHGAP1 KD cells, we could not obtain a sufficient number of viral particles for the immunization (Fig. 7A). The level of anti-IAV IgA was elevated by approximately 1.48-fold through immunization with viral particles produced from FIP5 KD cells compared with that of the control (Fig. 7E). In addition, the IgA antibody obtained from mice immunized with FIP5 KD virions neutralized the virus infectivity by 1.4- to 2.2-fold higher than that of the control virions (Fig. 7F). These findings indicate that the efficient budozone formation not only promotes the virus production but also increases the stability of viral particles and their protection against antibody responses.

## DISCUSSION

Although Rab11 REs are required for the apical transport of progeny vRNPs, Rab11 effectors regulating the vRNP transport remained controversial. Our proteomics analysis revealed that Rab11a interacts with class I FIPs (FIP1C, FIP2, and FIP5) in infected cells (Fig. 1D). We found that both FIP2 and FIP5 proteins are colocalized with vRNPs in ERCs and are required for the transport of vRNPs to the plasma membrane through the endocytic vesicles emanated from the ERCs (Fig. 2 and 3). It is reported that FIP2 forms a homodimer through the N-terminal C2 domain, which is highly conserved in class I FIPs (26, 49). Further, class I FIPs form heterodimers in the yeast two-hybrid system (50). On the basis of these results, FIP2 and FIP5 likely form a heterodimer and then regulate the kinesin-dependent anterograde transport of vRNPs. However, it is reported that transiently expressed full-length or truncated FIPs inhibited the ectopic localization of NP to the mitochondria expressing Rab11a fused to the mitochondria-targeting signal (29), suggesting that FIPs may compete with vRNPs for Rab11a binding. It is also proposed that KIF13, a kinesin family motor, binds to Rab11a and may directly regulate the intracellular transport of vRNPs (51). Further analysis is needed to understand the FIP-dependent and -independent pathways for the spatiotemporal regulation of vRNP transport.

In this study, the high-speed atomic force/fluorescence microscopy system enabled time-lapse imaging of budozone formation. The viral budozone was assembled in a lipid raft-dependent manner through the stabilization of actin filaments beneath the plasma membrane (Fig. 4 and 5). In COS-7 cells, we found that the viral budozone was surrounded by cortical actin filaments (Fig. 5A). Although the mesh size and filament orientation of cortical actin differ among the cell types used (52), such actin filaments, called actin vortices, are known to be highly stable but plastic higher-order structures (53). The cortical actin mesh is required for the formation of lipid rafts through the triggering of the liquid-ordered and liquid-disordered phase separation (54). Further, the lateral clustering of membrane proteins localized in the lipid raft is dependent on the contraction of cortical actin (40, 41). It has been reported that the mobility of HA decreases in regions with high densities of actin filaments and that the treatment with latrunculin A, a cortical actin-disrupting agent, dispersed the HA clusters on the plasma membrane (55). The reorganization of actin filaments is mainly regulated by Arp2/3, an actin-nucleating protein regulated by Cdc42, and MyoII, a molecular motor protein that reversibly cross-links actin filaments regulated by RhoA (56). Our findings revealed that the dynamics of cortical actin were stabilized by ARHGAP1, a negative regulator of Rho family proteins, upon IAV infection (Fig. 5C and D). Thus, it is possible that Rab11a REs mediate the apical transport of vRNPs with signal regulators, including cholesterol (35) and ARHGAP1, to trigger the budozone formation concomitantly with vRNP transport to the plasma membrane.

Although morphologic abnormalities were observed in virions produced from Rab11a KD cells, the biological significance has not been understood (57). In this study, we showed that the efficient budozone formation is crucial for the protease resistance and reduced antigenic potential of viral particles, possibly through the steric hindrance effect of clustered HA (Fig. 7D). HA consists of a globular head domain and a stalk domain. Most neutralizing antibodies recognize highly antigenic variable regions in the globular head domain, but not in other regions, including the stalk domain (58–60), possibly due to the fact that the other regions are hidden by clustering viral membrane proteins on the viral particles (61). Our findings contribute to the understanding of viral immune evasion strategy through the structural integrity of the viral particles, possibly by the efficient budozone formation.

## MATERIALS AND METHODS

### Affinity capture of biotinylated proteins.

At 2 h postinfection, myc-BirA*-Rab11a-expressing A549 cells were cultured with 50 μM biotin for 6 h. At 8 h postinfection, the cells were lysed in lysis buffer 1 (50 mM Tris-HCl, pH 7.4, 150 mM NaCl, 1% NP-40, 0.5% sodium deoxycholate, 2 mM MgCl_2_, 20 U Benzonase [Merck; catalog no. 70746], and complete protease inhibitor [Roche]) at 4°C for 5 min and then further lysed with lysis buffer 2 (50 mM Tris-HCl, pH 7.4, 150 mM NaCl, 1% NP-40, 0.5% sodium deoxycholate, 2 mM Tris(2-carboxyethyl)phosphine hydrochloride [TCEP], 0.2% SDS, and 2 mM EDTA). The soluble fractions were subjected to pulldown assays using streptavidin magnetic beads (TriLink; catalog no. M-1002). After being washed with wash buffers 1 to 4 (wash buffer 1, 50 mM Tris-HCl, pH 7.4, and 2% SDS; wash buffer 2, 20 mM Tris-HCl, pH 7.4, 500 mM NaCl, 1% Triton X-100, 0.1% sodium deoxycholate, and 1 mM EDTA; wash buffer 3, 10 mM Tris-HCl, pH 7.4, 250 mM LiCl, 1% NP-40, 0.1% sodium deoxycholate, and 1 mM EDTA; and wash buffer 4, 50 mM Tris-HCl, pH 7.4), the beads were incubated in an elution buffer (5 mM biotin, 150 mM NaCl, 2% SDS, and 5 mM TCEP) at 100°C for 5 min. The eluted proteins were subjected to LC-MS analysis using a Q Exactive HF-X mass spectrometer (Thermo Fisher Scientific). Gene ontology analysis of the identified proteins was performed using the DAVID database version 6.8.

### Intracellular localization of the viral genome and viral/cellular proteins.

Fluorescence *in situ* hybridization (FISH) assays and indirect immunofluorescence assays were carried out as previously described (62). The cells were fixed with 4% paraformaldehyde (PFA) for 10 min and permeabilized with 0.5% Triton X-100 in phosphate-buffered saline (PBS) for 5 min. After being incubated in PBS containing 1% bovine serum albumin for 30 min, the coverslips were incubated with each antibody for 1 h and then further incubated with Alexa Fluor 405-, 488-, and 568-conjugated secondary antibodies, respectively (Thermo Fisher Scientific). The permeabilization procedure was omitted for the staining of cell surface M2. FISH assays were performed after indirect immunofluorescence assays using an RNA probe complementary to the segment 7 virus genome. Images were acquired by confocal laser scanning microscopy (Carl Zeiss; LSM700) using an ×63 Apochromat objective (numerical aperture [NA], 1.4). The percentage of cytoplasmic vRNA signals colocalized with FIPs, excluding those localized around the ERC, the number of punctate ARHGAP1 signals in the cytoplasm, and the intensity of M2 signals on the cell surface, was calculated using Imaris software (Bitplane). Pearson's correlation coefficient of HA and M2 signals was calculated using the Fiji distribution of ImageJ software (63) with the Coloc 2 plugin.

### Gene silencing mediated by siRNA.

Short interfering RNAs (siRNAs) against *RAB11FIP1* (siRNA ID HSS149439), *RAB11FIP2* (1, siRNA ID HSS117714, and 2, siRNA ID HSS117713), *RAB11FIP5* (1, siRNA ID HSS178037, and 2, siRNA ID HSS119747), and *ARHGAP1* (1, siRNA ID HSS100669, and 2, siRNA ID HSS100671) genes were purchased (Thermo Fisher Scientific), and their transfection was carried out using Lipofectamine RNAi Max (Thermo Fisher Scientific) according to the manufacturer's protocol.

### Proximity ligation assays.

The quantification of budozone formation by proximity ligation assays (PLAs) was carried out as previously described (64). Briefly, cells were fixed with 4% PFA and then blocked with 1% milk for 30 min. The cells were incubated with mouse anti-HA antibody for 1 h and fixed again with 4% PFA. After permeabilization with 0.5% Triton X-100 for 5 min, the cells were further incubated with rabbit anti-M2 antibody for 1 h. PLA was carried out using a Duolink *in situ* PLA kit (Sigma) according to the manufacturer’s protocol. The number of PLA signals was measured using Imaris software.

### Transmission electron microscopy.

Preprocessing of the virions for TEM was carried out referring to a previously reported protocol (65). At 48 h postinfection of the WSN strain, the culture supernatants of A549 cells were centrifuged at 900 × *g* for 5 min at 4°C and then fixed with 2.5% glutaraldehyde at 4°C for 1 h followed by filtration using a 0.22-μm filter. The fixed viral particles were ultracentrifuged through a 20% (wt/vol) sucrose cushion at 78,400 × *g* for 1.5 h at 4°C. The purified virions were absorbed into hydrophilized acrylic-coated copper mesh grids (Okenshoji; catalog no. MMA-C10) and negatively stained with 2% phosphotungstic acid solution pH 5.8. Images were acquired using a JEM-1400 (Jeol). The diameter of viral particles and the number of spherical viral spike proteins with a diameter larger than 2.5 nm on the viral particles were measured manually using Fiji/ImageJ software (63).

### High-speed atomic force/fluorescence microscopic imaging.

A tip-scan high-speed atomic force microscope (AFM) equipped with an inverted confocal microscope (Olympus; BIXAM) was used as previously described (52, 66, 67). We used COS-7 cells as appropriate cells for the BIXAM imaging because they show high transfection efficiency and have a relatively flatter cell surface than epithelial cells. At 1 h posttransfection of HA-Venus and M2-mCherry, COS-7 cells were infected with the PR8 strain at an MOI of 10. At 12 h postinfection, images were acquired using BIXAM at 28°C with the phase modulation mode using an electron beam-deposited sharp cantilever tip with a spring constant of 0.05 nm^−1^ (USC-F0.8-k0.05; a customized cantilever purchased from NanoWorld). The confocal images and AFM images were simultaneously acquired at a scanning rate of 10 s/frame.

### Fluorescence recovery after photobleaching analysis.

A549 cells stably expressing Lifeact-TagGFP2 were infected with PR8 strain at an MOI of 10. At 12 h postinfection, FRAP analysis was performed with a 488-nm laser. The bleaching routine started with 2 prebleach scans followed by a bleaching scan. Then, the recovery of fluorescence was monitored every 300 ms for 40 s at 0.2% laser intensity. The fluorescence recovery data were normalized using data acquired from the nonbleached regions. To calculate the half-recovery time, the FRAP curves were fitted with the one-phase exponential equation using GraphPad Prism (version 7.03).

### Mouse models.

All the *in vivo* experiments were carried out according to the Guideline for Proper Conduct of Animal Experiments of the Science Council of Japan. The protocols for the animal experiments were approved by the Animal Care and Use Committee of the University of Tsukuba. Eight- to 10-week-old wild-type female C57BL/6J Jcl mice purchased from Clea Japan were used.

### Immunization of inactivated virions.

The viral particles (PR8) produced from FIP5 KD cells were concentrated by ultracentrifugation as described above. The purified virions were fixed with 0.1% formalin/PBS at 4°C for a week. Mice were intranasally immunized with inactivated virions equivalent to 1 μg of HA in the presence of 10 μg of poly(I:C) (InvivoGen; catalog code tlrl-pic) under anesthesia by intraperitoneal injection of pentobarbital sodium (Kyoritsu Seiyaku). The mice were reimmunized twice weekly, and the nasal washes were collected 1 week after the final immunization. The levels of total IgA and anti-IAV IgA antibodies were determined by ELISA as described previously (68).

### Neutralization assay.

Nasal wash samples containing equal amounts of total IgA in each sample were incubated with 4 × 10^4^ PFU of the PR8 strain at 37°C for 2 h. Then, the mixtures were inoculated into MDCK cells. At 4 h postinfection, the cells were fixed in 4% PFA, and the viral titers were determined by focus-forming assays with anti-NP antibody as described previously (69). The viral titer of viruses treated with nasal wash was represented as percentage of infectivity relative to that of untreated viruses.

### Statistical analysis.

The statistical significance of experimental results was determined by an unpaired two-tailed Student's *t* test or a one-way analysis of variance (ANOVA) with Tukey's test using GraphPad Prism (version 7.03). ns, not significant; *, *P* < 0.05; **, *P* < 0.01; ***, *P* < 0.001; ****, *P* < 0.0001.

10.1128/mbio.00721-22.8 TEXT S1 Supplemental materials and methods. Download Text S1, DOCX file, 0.04 MB. Copyright © 2022 Kuroki et al. 2022 Kuroki et al. https://creativecommons.org/licenses/by/4.0/ This content is distributed under the terms of the Creative Commons Attribution 4.0 International license.
