# Supplementary figures and images for: ARHGAP1 Transported with Influenza Viral Genome Ensures Integrity of Viral Particle Surface through Efficient Budozone Formation
Source: mBio. 2022 Apr 27;13(3):e00721-22. doi: 10.1128/mbio.00721-22 (PMC9239208; doi:10.1128/mbio.00721-22)

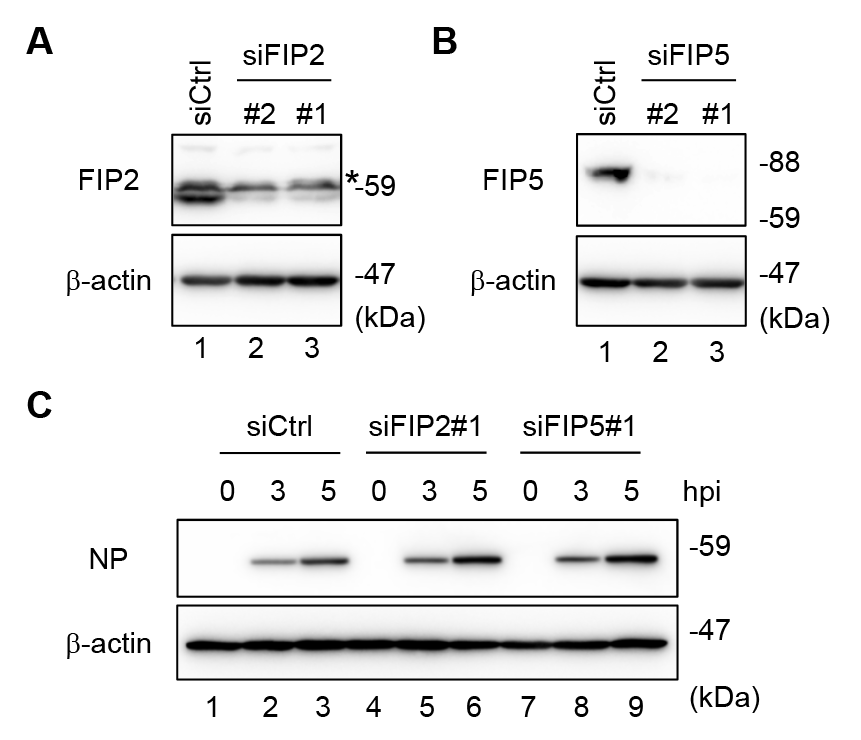

Supplement: FIG S1 [file mbio.00721-22-s0003.tif]

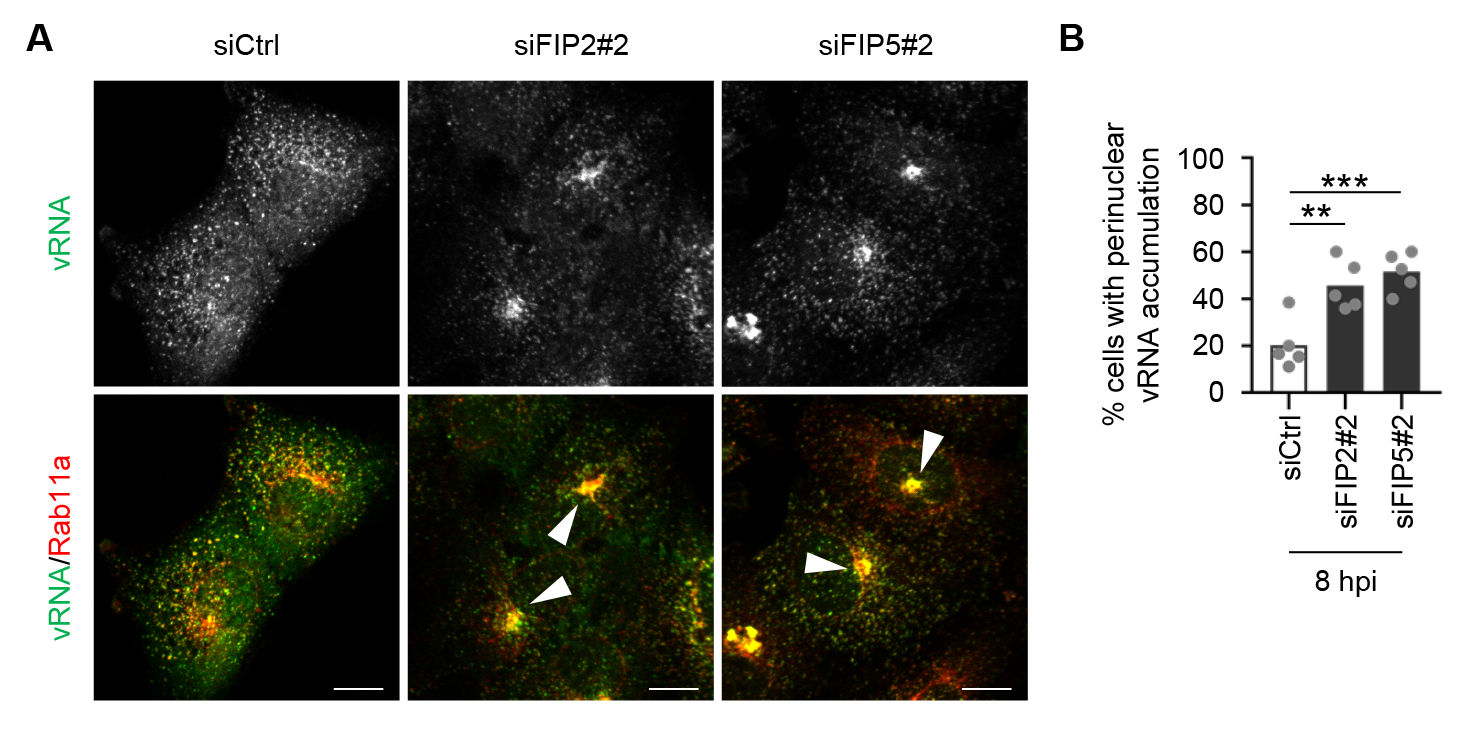

Supplement: FIG S2 [file mbio.00721-22-s0004.tif]

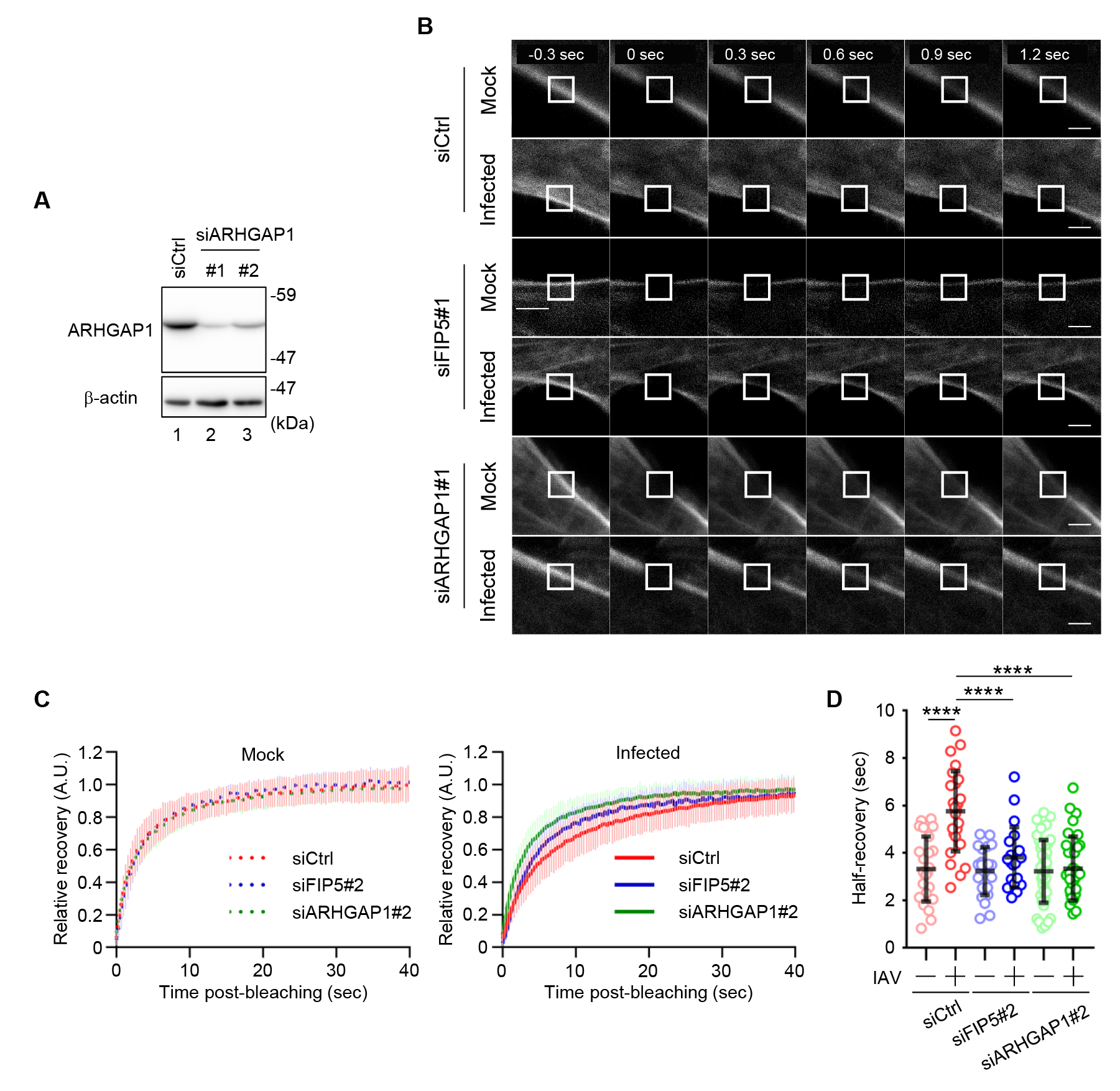

Supplement: FIG S3 [file mbio.00721-22-s0005.tif]

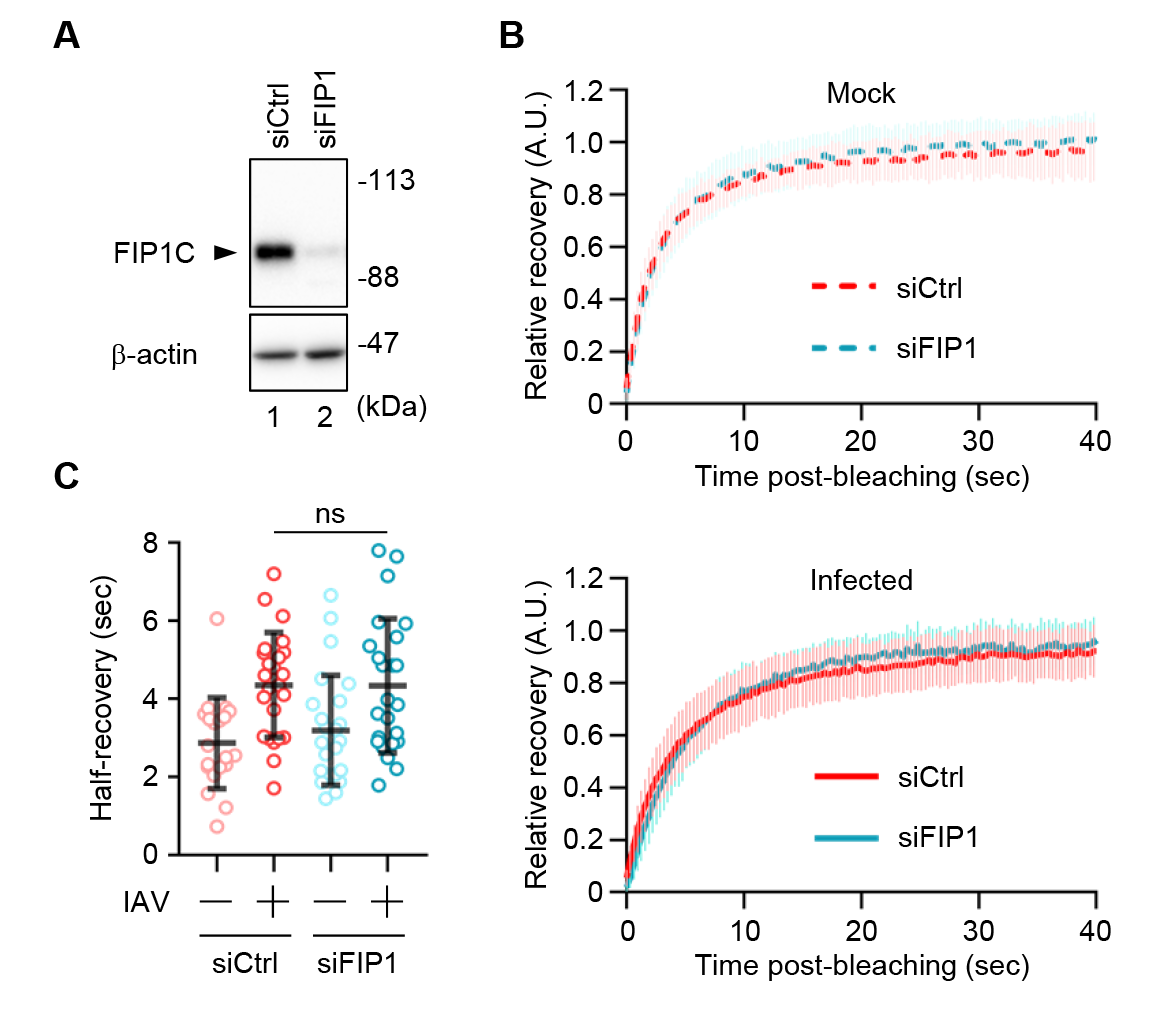

Supplement: FIG S4 [file mbio.00721-22-s0006.tif]

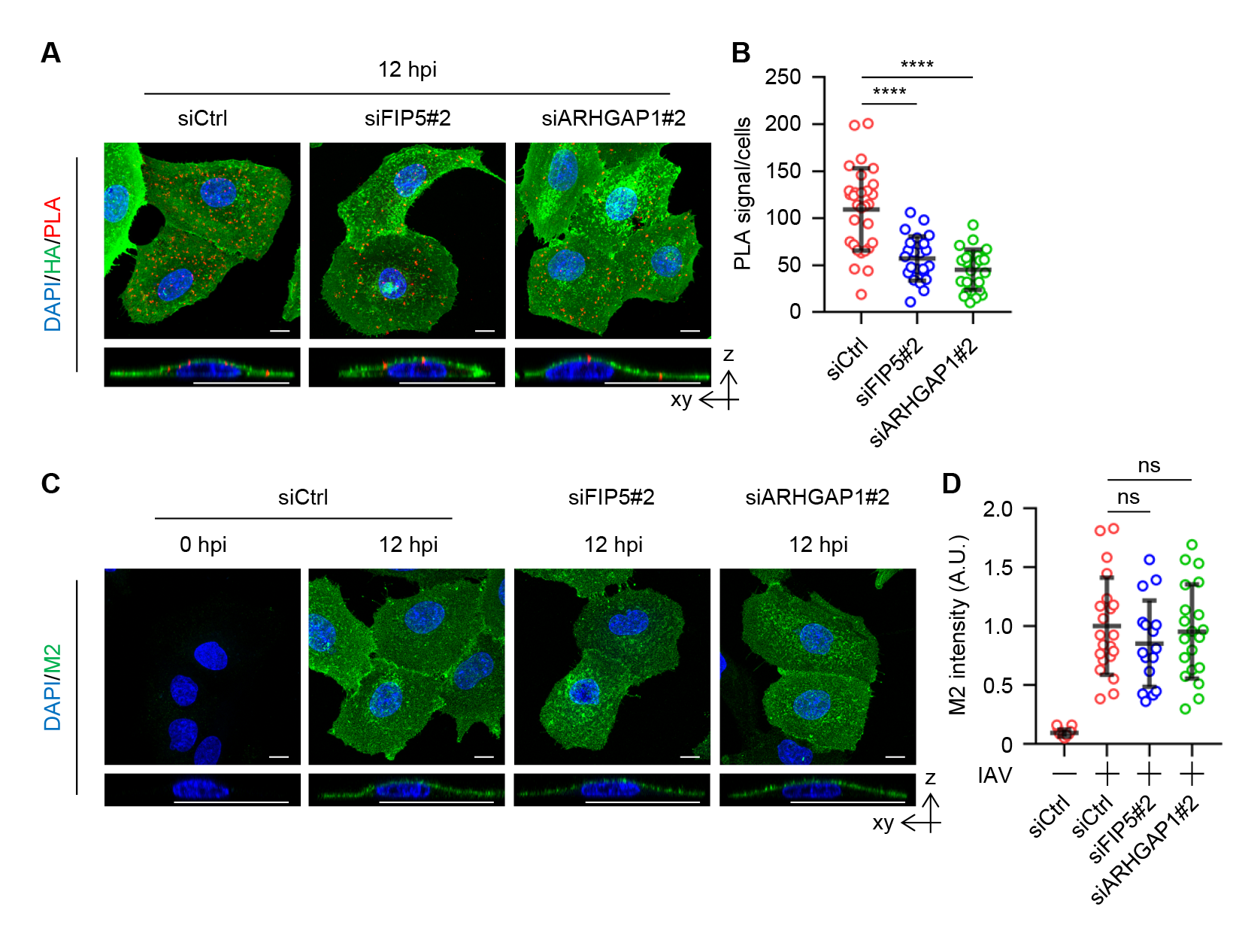

Supplement: FIG S5 [file mbio.00721-22-s0007.tif]

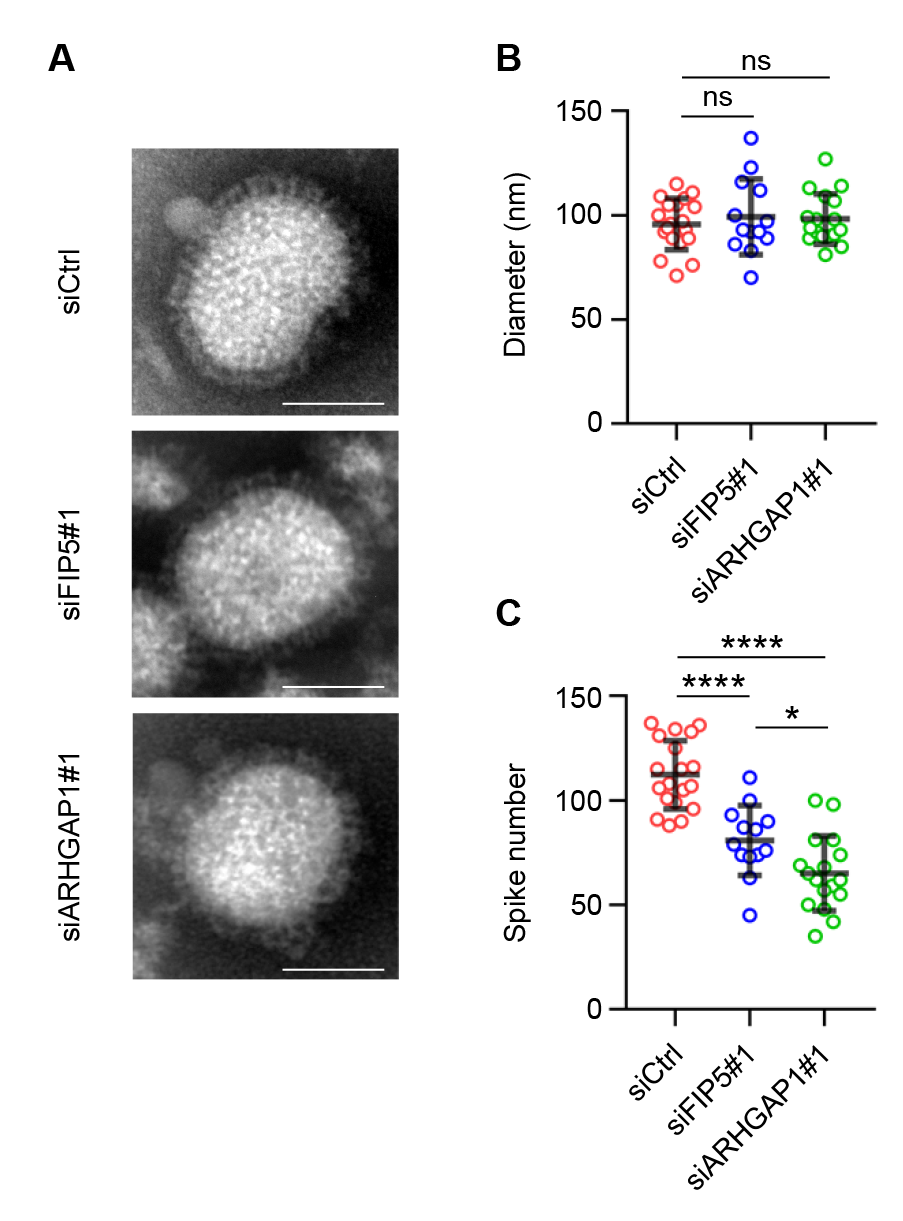

Supplement: FIG S6 [file mbio.00721-22-s0008.tif]
