# Supplementary material for: ARHGAP1 Transported with Influenza Viral Genome Ensures Integrity of Viral Particle Surface through Efficient Budozone Formation
Source: mBio. 2022 Apr 27;13(3):e00721-22. doi: 10.1128/mbio.00721-22 (PMC9239208; doi:10.1128/mbio.00721-22)
Supplement: TEXT S1 [file mbio.00721-22-s0001.docx]

**SUPPLEMENTAL MATERIAL**

**MATERIALS AND METHODS**

**Biological materials.**

Influenza A virus A/Puerto Rico/8/34 (PR8) and A/WSN/33 (WSN) strains were grown at 35.5°C for 48 h in the allantoic sacs of 11-day-old embryonated eggs, and the allantoic fluids were then collected and stored at -80°C until use. HEK293T, A549, COS-7, and MDCK cells were maintained in Dulbecco's minimal essential medium (DMEM) containing 10% fetal bovine serum and cultured at 37°C with 5% CO_2_. Rabbit polyclonal antibodies against NP, HA, and M1 were prepared as previously described (1). Other rabbit antibodies against myc tag (MBL; 562), β-actin (CST; 8547), FIP1 (Proteintech; 16778-1-AP), FIP2 (Proteintech; 18136-1-AP), FIP5 (Proteintech; 14595-1-AP), ARHGAP1 (Atlas Antibodies; HPA004689), and M2 (Abcam; ab56086) and mouse monoclonal antibodies against Rab11a (BD; 47/Rab11), FLAG tag (Sigma; F3165), and HA (TaKaRa; C179) were purchased. Cytochalasin D (Sigma; C8273) and chymotrypsin (Sigma; C4129) were purchased.

**Plasmid construction.**

For the construction of plasmids expressing FLAG-FIP2 and FLAG-ARHGAP1, A549 total RNA was reverse-transcribed as a template using the oligo(dT)_20_ primer, and the cDNAs were amplified with the primers 5’-CGCCGATATCGCCGCCACCATGGACTACAAGGATGACGACGACAAGATGATGCTGTCCGAGC-3’ and 5’-TGGGCGGCCGCCTATTAACTGTTAGAGAATTTGC-3’ for FIP2 and 5’-GGGCTCGAGGCCACCATGGATCCGCTCTCAGAGCTGCAGG-3’ and 5’-CCCGAATTCTCAGAGCCCGCTGGGGTCCGGGCTT-3’ for ARHGAP1, and then cloned into the pCAGGS-p7 and pCAGGS-FLAG plasmids (2), respectively. For the construction of pCAGGS-HA-Venus and pCAGGS-M2-mCherry plasmids, each cDNA was fused to the N terminus of the Venus or mCherry gene with a linker sequence, GGGGSGGGGS. The Venus and mCherry genes were obtained from pNPY-Venus-N1 (gifted by Dr A. Miyawaki, University of Tokyo) (3) and mCherry-Golgi-7 (Addgene #55052, gifted by Dr M. Davison, Florida State University), respectively. DNA transfection was carried out using the Neon transfection system according to the manufacturer's protocol (Thermo Fisher Scientific). For the construction of the lentivirus plasmid expressing the Lifeact-TagGFP2, the Lifeact-TagGFP2 cDNA from purchased pCAG-LifeAct-TagGFP2 plasmid (ibidi; 60106) was cloned into pCDH-CMV-MCS-EF1-Puro (System Biosciences; CD510B-1). For the construction of the lentivirus plasmid expressing the myc-tagged BirA^*^-fused Rab11a, Rab11a ORF was fused to the myc-BirA^*^ gene using pCDNA3.1-mycBioID (Addgene #35700, gifted by Dr K. Roux, University of South Dakota) (4) as a template, and the cDNA was then cloned into pCDH-CMV-MCS-EF1-Puro. The production of the lentivirus was carried out according to the manufacturer’s protocol.

REFERENCES

1. Kumakura M, Kawaguchi A, Nagata K. 2015. Actin-myosin network is required for proper assembly of influenza virus particles. Virology 476:141-150.

2. Murano K, Okuwaki M, Momose F, Kumakura M, Ueshima S, Newbold RF, Nagata K. 2014. Reconstitution of human rRNA gene transcription in mouse cells by a complete SL1 complex. J Cell Sci 127:3309-19.

3. Nagai T, Ibata K, Park ES, Kubota M, Mikoshiba K, Miyawaki A. 2002. A variant of yellow fluorescent protein with fast and efficient maturation for cell-biological applications. Nat Biotechnol 20:87-90.

4. Roux KJ, Kim DI, Raida M, Burke B. 2012. A promiscuous biotin ligase fusion protein identifies proximal and interacting proteins in mammalian cells. J Cell Biol 196:801-10.
